# Early Speech Development in Romanian Children with Cochlear Implants Assessed Using the LittlEARS^®^ Early Speech Production Questionnaire (LEESPQ)

**DOI:** 10.3390/audiolres15060172

**Published:** 2025-12-08

**Authors:** Alina Catalina Ivanov, Luminita Radulescu, Cristian Neagos, Sebastian Cozma, Corina Butnaru, Raluca Olariu, Petronela Moraru, Violeta Necula, Cristian Martu

**Affiliations:** 1 ENT Clinic Department, Grigore T. Popa University of Medicine and Pharmacy Iasi, 700115 Iasi, Romania; alina-catalina.bordeianu-ivanov@d.umfiasi.ro (A.C.I.); sebastian.cozma@umfiasi.ro (S.C.); butnaru.corina@umfiasi.ro (C.B.); raluca.olariu@umfiasi.ro (R.O.); moraru.petronela-ana-maria@d.umfiasi.ro (P.M.); martu.cristian@umfiasi.ro (C.M.); 2 Doctoral School of Medicine and Pharmacy, George Emil Palade University of Medicine, Pharmacy, Science, and Technology, 540139 Târgu Mureș, Romania; 3 ENT Clinic Department, Iuliu Hațieganu University of Medicine and Pharmacy Cluj, 400347 Cluj, Romania; violeta.necula@umfcluj.ro

**Keywords:** cochlear implant, early speech development, LEESPQ, auditory rehabilitation, parental questionnaire, language assessment, early intervention

## Abstract

**Objective:** The aim of the study was to evaluate the validity, clinical applicability, and developmental sensitivity of the Romanian LEESPQ in children with cochlear implants (CIs), by analyzing its association with age at implantation, duration of auditory experience, and implantation laterality, and by comparing the developmental trajectory with that of normal-hearing (NH) children. **Methods:** The study assesses the validity, reliability, and clinical sensitivity of the LEESPQ in pediatric cochlear implant users. Furthermore, it investigates the associations between total questionnaire scores and key clinical variables, including implantation laterality (unilateral versus bilateral), age at device activation, and duration of implant use. Forty-seven children with CIs (26 boys, 21 girls) were included, with implantation ages ranging from 9 months to 5 years. Of these, 21 received unilateral implants and 26 bilateral implants. Responses were analyzed both in relation to clinical variables and in comparison with available normative data from NH children, in order to delineate potential differences in linguistic developmental trajectories. **Results:** Findings suggest that the LEESPQ is a reliable and clinically valuable instrument for monitoring post-implant linguistic progress. It provides relevant insights into early auditory access, the linguistic environment within the family, and the development of early verbal production. Scores were significantly influenced by age at implantation and duration of auditory experience, confirming the role of early stimulation and neural plasticity in shaping speech development after cochlear implantation. **Conclusions:** The LEESPQ demonstrates strong clinical utility as a sensitive tool for monitoring early preverbal and verbal development in children with CIs. By capturing score variations associated with age at implantation, auditory experience, and implantation laterality, the questionnaire provides meaningful insights into early post-implant outcomes and supports individualized rehabilitation planning. These findings highlight the value of the LEESPQ for early outcome assessment in pediatric cochlear implant users.

## 1. Introduction

Cochlear implantation represents a crucial auditory intervention for children with severe to profound hearing loss, providing early access to sound and enabling the development of functional spoken language [1,2,3]. Given that auditory plasticity is maximal during the first years of life, rehabilitation outcomes largely depend on the timing of implantation, the duration of auditory exposure, and the quality of speech and language therapy [4,5,6,7,8]. This period of heightened neuroplasticity within the central auditory pathways allows rapid cortical reorganization and efficient integration of auditory input, thereby establishing the neural foundation for subsequent speech perception and language acquisition [5,6,7]. Monitoring language development during this critical period is essential for tailoring individualized therapeutic strategies [9,10].

Assessing early linguistic development in this age range is challenging because spontaneous speech is limited and direct testing often lacks sensitivity or feasibility. In this context, standardized parent-reported questionnaires provide a valuable alternative, capturing linguistic behaviors observed in the home environment [11,12,13]. Among these, the LittlEARS^®^ Early Speech Production Questionnaire (LEESPQ) was specifically designed to assess prelinguistic and early linguistic acquisition, covering the first 18 months of auditory experience. Comprising 27 binary (yes/no) items, the questionnaire evaluates reflexive behaviors, vocal reactions, emotional expressions, and the early emergence of expressive language [14,15,16,17].

The LEESPQ was originally developed in German and has since been adapted into multiple languages, including Turkish, Arabic, and Romanian, with all adaptations reliably demonstrating high internal consistency and strong construct validity [16,17,18]. The Romanian version, recently validated in a cohort of NH children, confirmed its psychometric robustness, including independence from gender and chronological age [18]. Nevertheless, most validation studies have been conducted in NH populations, and evidence regarding its applicability in children with CIs remains limited.

Compared with broader early communication tools such as the Preschool Language Scale (PLS) and the MacArthur–Bates Communicative Development Inventories (CDI), the LEESPQ targets only the preverbal and early verbal behaviors that emerge during the first months of auditory experience. This makes it particularly suitable for monitoring early speech-production development in children with cochlear implants, whose auditory age begins at device activation [11,19].

Children with CIs follow distinct language development trajectories compared to their NH peers, due to both delayed auditory exposure and differences in auditory processing [20,21,22]. While the LEESPQ has the potential to serve as an effective monitoring tool in this population, no datasets currently confirm its validity in the post-implant context. Furthermore, the lack of normative reference curves specific to cochlear-implanted children introduces significant uncertainty in clinical interpretation.

In Romania, the availability of standardized instruments for assessing early linguistic development remains extremely limited. Clinicians and therapists often rely on informal observation or partially validated scales, which inadequately address the lower age range or the specific features of post-implant rehabilitation [23,24]. Within this methodological gap, the LEESPQ stands out for its specificity to the first months of auditory exposure and its potential to guide early therapeutic intervention.

Moreover, involving parents in completing the questionnaire not only provides clinically relevant insights from the family environment but also increases parental awareness of their child’s progress, thereby strengthening therapeutic collaboration [25]. Through its simplicity in administration and interpretation, the LEESPQ emerges as an accessible tool for initial evaluation of language development in pediatric auditory rehabilitation.

In this context, the present study aimed to investigate early preverbal and verbal development in Romanian-speaking children with cochlear implants using the LEESPQ as an exploratory assessment tool. Specifically, the study examined how LEESPQ scores vary according to key clinical characteristics—age at implantation, duration of auditory experience, and implantation laterality—and compared these developmental trajectories with normative data from NH children. By analyzing subgroup differences and identifying factors associated with early speech-production outcomes, the study seeks to clarify the clinical utility of the LEESPQ in early post-implant monitoring and to identify patterns that may guide individualized rehabilitation strategies.

To date, no study has examined LEESPQ outcomes in Romanian-speaking children with cochlear implants. By providing the first developmental data in this population, the present study offers novel insights beyond existing international validations conducted in NH children and addresses an important clinical gap in early assessment tools available in Romania.

## 2. Materials and Methods

### 2.1. Study Design and Participants

This observational, cross-sectional, and comparative study was conducted on a cohort of 47 children (26 boys, 21 girls) with CIs. Of these, 21 children had unilateral and 26 had bilateral implantation. The age at implantation ranged from 8 months to 60 months. All participants were consecutively enrolled according to eligibility criteria and received standardized auditory rehabilitation and speech therapy programs in specialized centers. Participants were recruited from three leading university hospitals across Romania, ensuring coverage of diverse clinical approaches to pediatric cochlear implantation and rehabilitation.

Normative data used for comparison were obtained from the previously published Romanian LEESPQ validation study, which included 148 NH children and established the developmental reference curve for typical auditory and speech development [18]. These normative data were used in the present study to compare the estimated developmental trajectory of LEESPQ scores in children with CIs against age-equivalent auditory experience in NH children, including comparisons of fitted regression curves and subgroup analyses across matched audiological age intervals (0–6, 7–12, and 13–18 months).

### 2.2. Inclusion and Exclusion Criteria

Children were eligible if they met the following criteria: (1) audiologically confirmed severe-to-profound hearing loss; (2) cochlear implantation performed between 9 months and 5 years of age; (3) regular participation in speech therapy sessions as confirmed by parents; (4) parental/legal guardian consent and signed informed consent; (5) predominant exposure to a native-language (Romanian) environment; and (6) absence of major neurological or cognitive impairments affecting language development.

Exclusion criteria included: (1) severe cognitive or neurological disorders interfering with language acquisition; (2) major associated comorbidities (e.g., complex genetic syndromes, multiple disabilities); and (3) lack of informed consent from parents/legal guardians.

### 2.3. Assessment Tool

Early speech development was assessed using the LittlEARS^®^ Early Speech Production Questionnaire (LEESPQ), a standardized instrument designed to monitor preverbal and early verbal achievements during the first 18 months of auditory exposure [14]. The questionnaire includes 27 binary items (“yes”/”no”), covering domains of reflexive and prelinguistic behaviors, vocal and emotional responses, and the onset of expressive language.

Originally developed and validated in German, the LEESPQ has since been translated and culturally adapted into several languages, including Turkish, Arabic, and Romanian, with all adaptations showing high internal consistency (Cronbach’s α > 0.85) and strong construct validity. The Romanian version was recently validated in a cohort of 148 NH children, confirming excellent reliability, independence from gender and chronological age, and a developmental trajectory comparable to that of the original German version. This validation supports the use of the Romanian LEESPQ as a psychometrically sound tool for both research and clinical applications, including its adaptation to pediatric populations with CIs.

The LEESPQ is a copyrighted instrument developed by MED-EL. It is available free of charge for clinical and research use and can be obtained upon request from the manufacturer. Clinicians and researchers may access the questionnaire and related materials through the official LittlEARS program by contacting MED-EL (Innsbruck, Austria) or through the LittlEARS resources webpage (https://www.medel.com/) (accessed on 1 October 2025) Permission for use is required for distribution and adaptation.

### 2.4. Procedure

The questionnaire was administered through structured telephone interviews with parents or legal guardians, conducted by audiologists and speech therapists from multiple Romanian medical centers, as part of routine post-implant follow-up evaluations. Parents provided responses based on observed child behaviors in the home environment, with no time restrictions, thereby ensuring accuracy and ecological validity.

Before administration, parents received standardized instructions and examples to ensure consistent understanding of each questionnaire item while minimizing response bias.

To minimize interviewer bias, all interviewers used a standardized script, received uniform training, and followed identical instructions across centers.

In addition to LEESPQ scores, clinical data were collected: age at implantation (months), duration of implant use (months since activation), and type of implantation (unilateral/bilateral). All assessments were carried out under standardized conditions, and all data were anonymized prior to analysis.

### 2.5. Ethical Considerations

The study was conducted in accordance with the ethical principles of the Declaration of Helsinki and applicable national regulations on research involving human subjects. Ethical approval was obtained from the institutional review boards of the participating centers. Parents/legal guardians received detailed information regarding the purpose and procedures of the study and provided written informed consent. Participation was voluntary, with the right to withdraw at any time without any consequences. Data confidentiality was maintained through anonymization using unique participant codes and secure storage accessible only to the research team.

## 3. Results

### 3.1. Statistical Analysis

Internal consistency of the LEESPQ within the CI cohort was examined using Cronbach’s α. The 27 items demonstrated good reliability, with Cronbach’s α = 0.799, indicating that the questionnaire items function cohesively in this clinical population.

The relationship between LEESPQ total scores and time since cochlear implantation was examined in order to evaluate the estimated developmental trajectory of preverbal and early verbal auditory skills relative to auditory experience. The analysis demonstrated a clear positive trend, with scores increasing as the duration of implant use lengthened.

Pearson’s correlation coefficient indicated a moderate-to-strong positive association between the two variables (r = 0.58, *p* < 0.0001), while Spearman’s rank correlation produced comparable results (ρ = 0.52, *p* < 0.001), thereby supporting the robustness of the finding across parametric and non-parametric approaches. These results suggest that children with longer auditory experience following cochlear implantation tend to achieve higher LEESPQ scores, reflecting improvements in early speech production and prelinguistic auditory behaviors over time.

**Figure 1:**
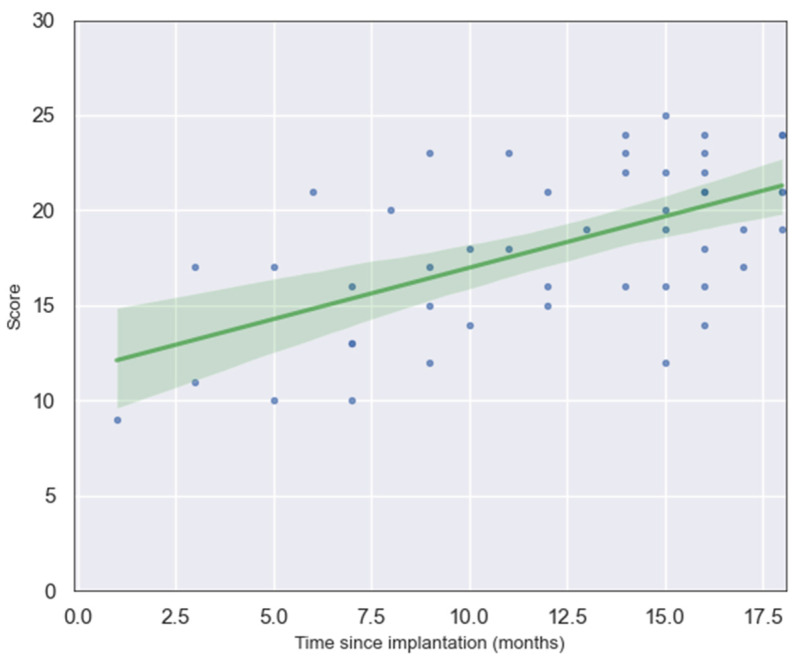
Scatterplot showing the relationship between LEESPQ total score and time since cochlear implantation. Each point (blue dot) represents an individual child’s score, and the green line depicts the fitted linear regression with its 95% confidence interval.

A linear regression analysis further confirmed this association, revealing that time since implantation significantly predicted LEESPQ score (β = 0.54, SE = 0.11, t = 4.88, *p* < 0.001). The 95% confidence interval for this coefficient ranged from 0.32 to 0.76, indicating a robust positive effect. The regression intercept was 11.57 (SE = 1.44, 95% CI: 8.60–14.41). The model accounted for approximately 34% of the variance in total scores (R^2^ = 0.34). The regression equation is expressed as:

y = 11.57 + 0.54 x(1)

where x is time since implantation in months. This finding demonstrates that, on average, each additional month of auditory experience with a cochlear implant is associated with an increase of approximately half a point in the child’s LEESPQ score. This strong linear relationship highlights the steady and predictable nature of early speech development following cochlear implantation, suggesting that LEESPQ scores can serve as a practical quantitative indicator of linguistic progression during the first stages of auditory rehabilitation. A scatter plot of the raw data and the generated norm curve is shown in Figure 1.

The analysis of the relationship between LEESPQ scores and age at implantation revealed a negative correlation. Children who received their cochlear implant at a younger age tended to obtain higher scores compared to those implanted later.

Pearson’s correlation analysis showed a moderate negative correlation (r = −0.44, *p* = 0.0018), and this result was corroborated by Spearman’s rank correlation (ρ = −0.49, *p* < 0.001). These findings confirm that later age at implantation is associated with reduced performance in early speech production and prelinguistic auditory skills as assessed by the LEESPQ.

Taken together, these results highlight that early implantation and longer auditory experience are key factors linked to improved preverbal and early verbal auditory outcomes in children with CIs, as measured by the LEESPQ.

To further explore the influence of age at implantation on early speech production outcomes, participants were divided into two groups: those implanted early (<24 months) and those implanted later (≥24 months).

**Figure 2:**
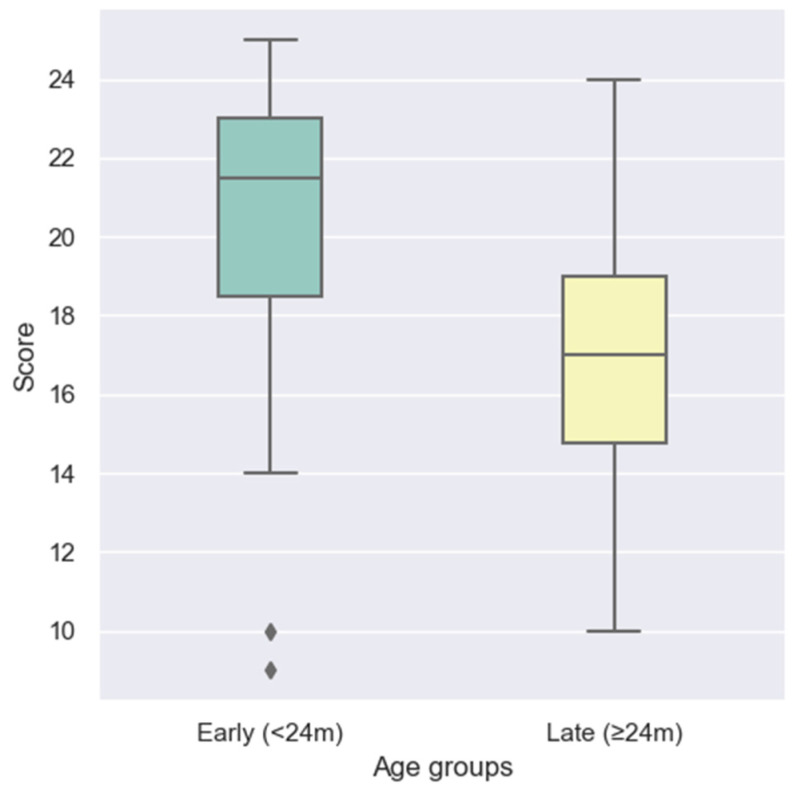
Comparison of LEESPQ scores between early-implanted (<24 months) and late-implanted (≥24 months) children. Diamond-shaped points represent outliers identified using the 1.5× IQR criterion.

Comparative analysis revealed a significant advantage for children receiving their implant before the age of two years. As illustrated in the boxplot in Figure 2, the early implantation group achieved higher LEESPQ scores compared to the late implantation group. A Mann–Whitney U test confirmed that this difference was statistically significant (U = 424.5, *p* = 0.0025).

**Figure 3:**
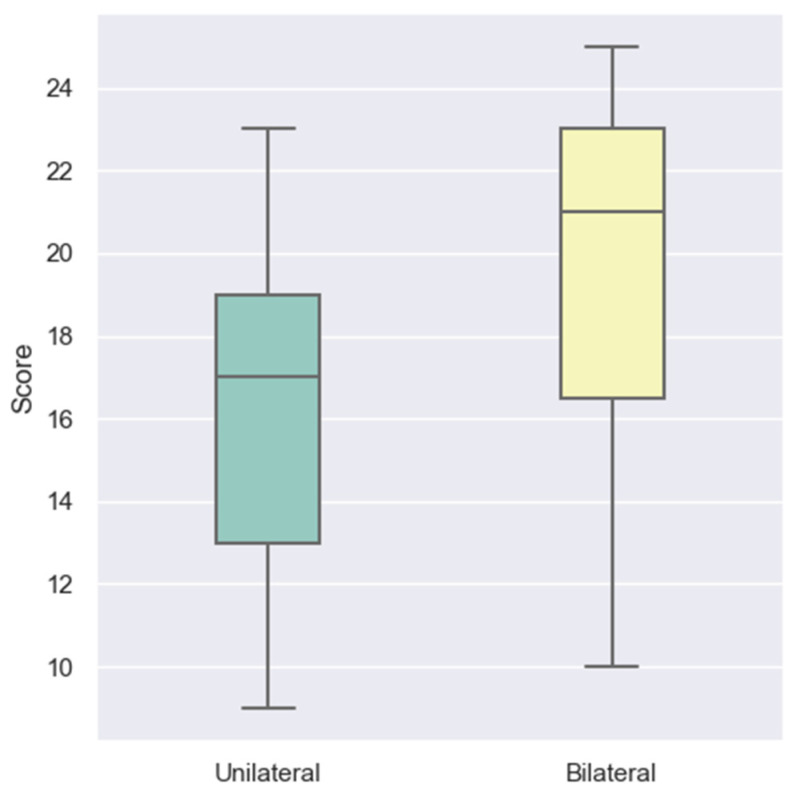
Distribution of LEESPQ total scores in children with CIs, stratified by implantation laterality.

The impact of implantation laterality on LEESPQ outcomes was assessed by comparing children with unilateral cochlear implantation to those with bilateral implantation. The boxplot from Figure 3 suggests that bilateral recipients tend to achieve higher scores than unilateral recipients.

This observation was supported by statistical analysis. The Mann–Whitney U test revealed a significant difference between groups (U = 156.0, *p* = 0.0081), with children receiving bilateral implants demonstrating superior LEESPQ performance. These findings indicate that access to binaural auditory input may facilitate more efficient speech perception and early verbal production by enhancing auditory spatial processing and signal integration during the early stages of rehabilitation.

Normality of continuous variables was assessed using the Shapiro–Wilk test. Given the exploratory nature of the study, no corrections for multiple comparisons were applied; this approach is consistent with the aim of generating preliminary insights into subgroup differences. Potential confounding factors such as parental education level or intensity of speech therapy were considered; however, these variables were not included in the models due to incomplete reporting across participating centers.

### 3.2. Comparison with Normal Children

To examine developmental trajectories in early speech production, LEESPQ scores were compared between children with normal hearing and those with CIs, using audiological age as a common metric. For NH children, chronological age was considered equivalent to auditory experience, while for implanted children, time since implantation was used as the measure of auditory age.

The regression analysis for NH children followed a quadratic model, consistent with previous validation studies [18]. The fitted equation was:

y = −0.04x^2^ + 1.48x + 7.47 (2)

where x represents age in months. This curve reflects an inferred trend of rapid increase in preverbal and early verbal auditory skills during the first year of life, followed by a gradual plateau approaching the maximum LEESPQ score.

In contrast, scores in the implanted group were best described by a linear regression model:

y = 11.57 + 0.54x(3)

where x represents time since implantation in months.

**Figure 4:**
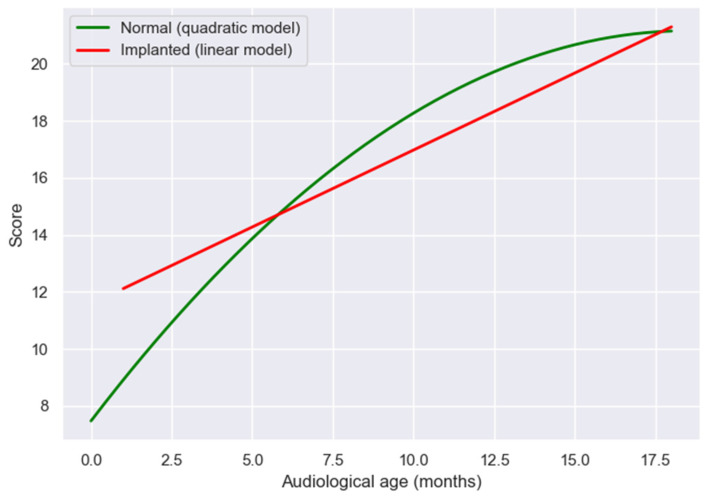
Comparison of LEESPQ derived developmental patterns between NH and cochlear-implanted children, using audiological age as the reference metric.

When the two regression functions were compared graphically, implanted children demonstrated initial scores below those of NH peers at equivalent early audiological ages, as illustrated in Figure 4. However, the estimated progression patterns showed progressive convergence over time, with implanted children approaching the estimated developmental trajectory of NH children at approximately 18 months of auditory experience. This convergence pattern suggests that, once consistent auditory input is established through cochlear implantation, the pace of linguistic acquisition follows a near-typical trajectory, reflecting the capacity of the developing auditory system to reorganize and adapt to new sensory input.

**Figure 5:**
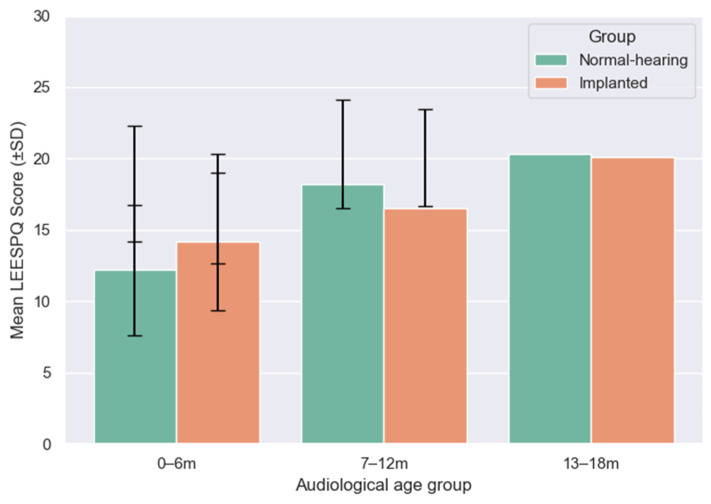
Mean LEESPQ scores (±SD) for NH and cochlear-implanted children across matched audiological age ranges (0–6, 7–12, and 13–18 months).

To enable direct comparison of developmental outcomes, LEESPQ scores of children with CIs were contrasted with those of NH children across matched audiological age ranges (0–6, 7–12, and 13–18 months), as illustrated in Figure 5.

In the 0–6 months range, the mean LEESPQ score of the implanted group (14.2 ± 4.8) was slightly higher than in the NH group (12.2 ± 4.6). However, this difference did not reach statistical significance (Mann–Whitney U test, *p* = 0.44), and the effect size was small (rank-biserial correlation = +0.19).

For the 7–12 months range, NH children scored higher on average (18.2 ± 4.1) compared to implanted peers (16.5 ± 3.8). The difference approached statistical significance (*p* = 0.089) and showed a medium effect size (−0.27), suggesting a trend towards lower scores in implanted children during this interval.

By the 13–18 months range, mean scores of implanted children (20.1 ± 3.4) closely approximated those of NH peers (20.3 ± 3.8), with no significant difference observed (*p* = 0.76). The effect size was negligible (−0.04), indicating convergence of the two groups at later stages of auditory experience.

Overall, these comparisons highlight a dynamic estimated developmental trajectory: implanted children show comparable or slightly higher scores than NH peers in the earliest months, exhibit a transient lag during the intermediate period, and ultimately converge toward typical developmental levels by 18 months of auditory experience.

## 4. Discussion

The present study provides novel evidence regarding the applicability of the LittlEARS^®^ Early Speech Production Questionnaire (LEESPQ) in Romanian children with CIs. The findings demonstrate that the LEESPQ is a reliable and clinically informative tool for monitoring preverbal and early verbal development after implantation, with results highlighting the influence of key clinical variables such as age at implantation, duration of auditory experience, and type of implantation. The originality of this work lies in its focus on cochlear-implanted children, a population not included in previous Romanian or international LEESPQ validation studies. By analyzing subgroup differences and developmental trajectories, the study provides the first evidence on how early speech-production abilities evolve in Romanian-speaking CI users. These insights have direct clinical relevance, offering clinicians an accessible tool for early monitoring, supporting the harmonization of rehabilitation protocols, and helping identify children who may benefit from intensified therapeutic support.

Consistent with previous literature, the data confirm that early implantation is associated with higher LEESPQ scores [3,4,5,6]. Children implanted before 24 months achieved significantly higher LEESPQ scores compared to those implanted later, corroborating prior studies emphasizing the benefits of implantation within the sensitive period of auditory plasticity [5,6,7]. These results further support international guidelines advocating for early diagnosis and intervention within the first years of life.

Recent literature further supports the patterns observed in the present study regarding early language development after cochlear implantation. Arras et al. (2023) demonstrated that children with pre-lingual single-sided deafness who received a cochlear implant achieved early narrative and verbal short-term memory abilities comparable to their NH peers, underscoring the benefits of timely auditory intervention [26]. Similarly, Yin et al. (2022) reported that Mandarin-speaking children with simultaneous bilateral cochlear implants showed faster early language gains compared with unilateral recipients, highlighting the advantages of bilateral auditory stimulation during the initial stages of language acquisition [27]. These recent findings are consistent with our subgroup results and reinforce the clinical importance of early implantation, enriched auditory exposure, and structured rehabilitation programs in optimizing early speech-production outcomes in children with cochlear implants.

The duration of implant use was also a significant predictor of LEESPQ performance. Longer auditory experience was positively correlated with higher scores, underscoring the cumulative effect of consistent auditory input on early speech acquisition. This observation aligns with the developmental trajectories reported in longitudinal studies such as the LOCHI project, which similarly demonstrated that extended hearing experience promotes more advanced language outcomes [8,13].

With respect to implantation laterality, children with bilateral devices outperformed their unilateral peers. This finding reinforces existing evidence that bilateral auditory stimulation facilitates more natural binaural hearing, improves speech-in-noise perception, and enhances language development. Although not all studies have consistently demonstrated large differences between unilateral and bilateral users, our results suggest that bilateral implantation provides measurable benefits in the first phases of auditory and linguistic development.

Comparison with normative data from NH children provided further insights into developmental trajectories. At equivalent early auditory ages, children with CIs initially scored below NH peers, reflecting delayed access to auditory input prior to implantation. [18,20]. However, with increasing auditory experience, scores converged toward normative values by 18 months of “audiological age.” This convergence suggests that, while implantation cannot fully replicate typical auditory development, early and sustained auditory exposure allows children to approach age-appropriate language milestones. This insight highlights the remarkable capacity of the developing auditory cortex to reorganize in response to electrical stimulation, underscoring the central role of neuroplasticity in achieving functional language outcomes. Moreover, the LEESPQ’s sensitivity to these progressive changes suggests its potential use as a marker of auditory cortical adaptation in early rehabilitation. Importantly, our findings also suggest a transient lag during the 7–12 month range, potentially reflecting the additional effort required for adaptation to electrical stimulation and integration of auditory input.

The difference in curve shape between the two groups is noteworthy. In NH children, the quadratic trajectory reflects the well-documented rapid acceleration of early speech-production behaviors during the first months of natural auditory exposure, followed by a plateau as skills become consolidated. In contrast, the linear pattern observed in the CI group likely reflects their delayed onset of auditory stimulation and a more gradual, steady rate of improvement. Rather than exhibiting the early peak of rapid growth seen in typical development, children with cochlear implants appear to follow a catch-up process characterized by continuous incremental gains as auditory experience accumulates. This interpretation aligns with previous literature describing more gradual early language acquisition after implantation.

The use of a parent-reported measure such as the LEESPQ offers several advantages. It captures behaviors observed in naturalistic environments, reduces the limitations of clinical testing in very young children, and actively involves parents in the rehabilitation process. Moreover, its simplicity and standardization allow for repeated monitoring across time, making it a practical instrument for clinicians and therapists in Romania, where validated early language assessment tools are scarce. Furthermore, integrating the LEESPQ into standard post-implant rehabilitation protocols could support more consistent national monitoring practices and help optimize early intervention programs for children with hearing loss. In the broader context of pediatric cochlear implant assessment, the LEESPQ represents the earliest stage of developmental monitoring, capturing preverbal and emerging verbal behaviors during the first months of auditory experience. At later ages, complementary tools such as the Meaningful Auditory Integration Scale (MAIS) and the Meaningful Use of Speech Scale (MUSS) become more appropriate [28,29]. The MUSS, typically used in children from approximately three years of age onward, evaluates spontaneous speech production, speech intelligibility, and functional communicative use of spoken language. Together, these instruments form a sequential continuum—LEESPQ for early speech-production milestones, MAIS for functional auditory integration, and MUSS for expressive communication—allowing clinicians to monitor auditory and speech development across the full pediatric age span.

Although the Romanian LEESPQ was validated in NH children, parental interpretation in CI families may differ, and the use of a parent-report tool introduces potential subjective bias linked to variations in auditory experience and home environments.

Despite these strengths, several limitations should be acknowledged. First, the cross-sectional design precludes the examination of longitudinal developmental changes within individuals. Second, although the sample size was adequate for the analyses conducted, larger multicenter studies would provide stronger generalizability and enable subgroup analyses (e.g., by etiology of hearing loss, device manufacturer, socioeconomic status or participation in speech therapy sessions, which may exert a significant influence on early verbal outcomes).

## 5. Conclusions

The present study investigated early preverbal and verbal developmental outcomes in Romanian-speaking children with CIs using the LittlEARS^®^ Early Speech Production Questionnaire (LEESPQ) and evaluated the questionnaire’s applicability and clinical sensitivity within this population. The findings demonstrate that the LEESPQ is capable of capturing meaningful variability in early speech-production skills and responds sensitively to key clinical factors such as age at implantation, duration of auditory experience, and implantation laterality.

Children implanted at a younger age demonstrated significantly higher LEESPQ scores, emphasizing the critical importance of early auditory stimulation within the sensitive period of neuroplasticity. Similarly, longer duration of implant use was strongly correlated with higher scores, emphasizing the progressive benefits of continuous auditory exposure on speech and language development. Bilateral cochlear implantation was also associated with superior outcomes compared to unilateral implantation, supporting the role of binaural hearing in enhancing speech perception, sound localization, and early verbal abilities.

When compared to NH peers, children with CIs initially displayed lower LEESPQ scores, reflecting the delay in auditory access typical of this population. However, as auditory experience accumulated, their scores showed an apparent trend toward normative trajectories, with no significant differences observed after approximately 18 months of auditory exposure.

Overall, the results indicate that the LEESPQ is a sensitive and clinically informative instrument for the early assessment of preverbal and verbal abilities in children with CIs. By capturing developmental differences linked to age at implantation, auditory experience, and implantation laterality, the questionnaire provides meaningful insights that can support individualized rehabilitation strategies. Its structured, parent-reported format enables routine monitoring during the earliest phases of auditory development, a period in which standardized assessment tools remain limited. The LEESPQ may also support parental counseling by helping caregivers understand their child’s early auditory and speech-production progress, thereby facilitating timely engagement in rehabilitation strategies.

Future studies should further validate these findings in larger and longitudinal cohorts and investigate the extent to which early LEESPQ performance predicts later speech, language, and communication outcomes. Cross-cultural validation may help determine whether early LEESPQ patterns are consistent across different linguistic and cultural settings.

## Data Availability

The original data presented in the study are openly available in FigShare at doi:10.6084/m9.figshare.30813314. The anonymized dataset generated and analyzed in this study will be made openly accessible in a public online repository upon publication of the article.
