# Correction: Ivanov et al. Early Speech Development in Romanian Children with Cochlear Implants Assessed Using the LittlEARS^®^ Early Speech Production Questionnaire (LEESPQ). *Audiol. Res.* 2025, *15*, 172

**DOI:** 10.3390/audiolres16020030

**Published:** 2026-02-25

**Authors:** Alina Catalina Ivanov, Luminita Radulescu, Cristian Neagos, Sebastian Cozma, Corina Butnaru, Raluca Olariu, Petronela Moraru, Violeta Necula, Cristian Martu

**Affiliations:** 1 ENT Clinic Department, Grigore T. Popa University of Medicine and Pharmacy Iasi, 700115 Iasi, Romania; alina-catalina.bordeianu-ivanov@d.umfiasi.ro (A.C.I.); sebastian.cozma@umfiasi.ro (S.C.); butnaru.corina@umfiasi.ro (C.B.); raluca.olariu@umfiasi.ro (R.O.); moraru.petronela-ana-maria@d.umfiasi.ro (P.M.); martu.cristian@umfiasi.ro (C.M.); 2 Doctoral School of Medicine and Pharmacy, George Emil Palade University of Medicine, Pharmacy, Science, and Technology, 540139 Târgu Mureș, Romania; 3 ENT Clinic Department, Iuliu Hațieganu University of Medicine and Pharmacy Cluj, 400347 Cluj, Romania; violeta.necula@umfcluj.ro

## Error in Affiliation

In the published paper [1], there was an error regarding the affiliation for Cristian Neagos. Regarding affiliation 2, the updated affiliation should be: Doctoral School of Medicine and Pharmacy, George Emil Palade University of Medicine, Pharmacy, Science, and Technology, 540139 Târgu Mureș, Romania. The authors state that the scientific conclusions are unaffected. This correction was approved by the Academic Editor. The original publication has also been updated.
